# Circular RNA hsa_circ_0072309 promotes tumorigenesis and invasion by regulating the miR-607/FTO axis in non-small cell lung carcinoma

**DOI:** 10.18632/aging.202856

**Published:** 2021-04-20

**Authors:** Wei-Lie Mo, Li-Jian Deng, Yun Cheng, Wen-Jun Yu, Yan-Hua Yang, Wei-Dong Gu

**Affiliations:** 1 Department of General Surgery, Changzhou Seventh People's Hospital, Changzhou 213011, Jiangsu, China; 2 Department of General Surgery, Changzhou Geriatric Hospital Affiliated to Soochow University, Changzhou 213011, Jiangsu, China; 3 Department of Oncology, Changzhou Seventh People's Hospital, Changzhou 213011, Jiangsu, China; 4 Department of Traumatology, Changzhou Seventh People's Hospital, Changzhou 213011, Jiangsu, China

**Keywords:** NSCLC, hsa_circ_0072309, miR-607, FTO, sponge effect

## Abstract

Emerging evidence has demonstrated that circular RNAs (circRNAs) are abnormally expressed in non-small cell lung carcinoma (NSCLC). However, the contributions of circRNAs to the tumorigenesis of lung adenocarcinoma (LUAD), one of the subtypes of NSCLC, remain unclear. Based on a microarray assay, we found that hsa_circ_0072309 was significantly upregulated in NSCLC compared with matched normal samples. Moreover, functional experiments demonstrated that hsa_circ_0072309 promotes the proliferation, migration, and invasion of NSCLC cells. *In vitro* precipitation of circRNAs, luciferase reporter assays, and biotin-coupled microRNA capture assays were carried out to investigate the mechanisms by which hsa_circ_0072309 regulates NSCLC. Through the above work, we found that hsa_circ_0072309 interacted with miR-607 via its miRNA response element to upregulate the expression of FTO, an m6A demethylase and downstream target of miR-607, thus promoting tumorigenesis of NSCLC. In total, our findings indicated the oncogenic role of hsa_circ_0072309 in NSCLC and provide a potential target for treatment.

## INTRODUCTION

Cancer is a major public health issue worldwide, and lung cancer is one of the most common types. Studies have shown that the global incidence and mortality of lung cancer have reached 11.6% and 18.4%, respectively. More than 80% of all lung cancer cases are non-small cell lung cancer, which is the main cause of lung cancer-specific mortality [1, 2]. However, elucidation of the mechanisms underlying lung cancer progression and development of treatment strategies are urgently needed. Recent progress in RNA research has led to the identification of noncoding RNAs (ncRNAs) that are involved in many kinds of biological processes, including the formation and progression of cancers. Once regarded as ‘noise’ or ‘junk’ of the human transcriptome, microRNAs (miRNAs) and lncRNAs have been shown to play critical roles in the regulation of proliferation, apoptosis, invasion and other processes in lung cancer, revealing a novel dimension of cancer biology [3, 4].

Circular RNAs are a novel category of endogenous noncoding RNAs formed by noncanonical splicing of exonic and intronic sequences [5, 6]. Distinct from regular linear RNAs, circRNAs are covalently closed without 5' and 3' ends [7, 8], which makes them more resistant to exonucleases. Recently, circRNAs have been shown to have important effects in some diseases, including human cancers, because of their conservation, abundance and tissue specificity [6, 9, 10]. Mechanistically, circRNAs participate in multiple cellular events because they can be used as scaffolds in the assembly of protein complexes, sequester proteins from their native subcellular locations, and regulate mRNA alternative splicing. Importantly, a portion of circRNAs, which have miRNA response elements (MREs), can act as miRNA sponges and thereby affect downstream target genes of miRNAs [9–14]. For instance, circFoxo3 promoted tumor cell apoptosis to inhibit tumor growth in breast cancer [15–17]. Circ-ZKSCAN1 inhibited the progression of bladder cancer via the miR-1178-3p/p21 axis and was a prognostic factor for recurrence [18]. In contrast to circFoxo3 and circ-ZKSCAN1, circRNA MTO1 sponged miR-9 and upregulated its target p21 to suppress HCC progression [19]. Some studies have also demonstrated that circRNAs participate in the progression of NSCLC by sponging conserved miRNAs. Qiu et al. revealed that circFGFR3 could promote NSCLC cell invasion and proliferation by competitively binding miR-22-3p to increase the expression of galectin-1 (Gal-1), p-AKT, and p-ERK1/2 [20]. Hsa_circ_100395 was shown to inhibit the progression of lung cancer through the miR-1228/TCF21 axis [21]. However, the critical roles of circRNAs in NSCLC remain largely unclear.

In this study, we found that hsa_circ_0072309 was upregulated in NSCLC tissues and cells. Functionally, we found that si-hsa_circ_0072309 suppressed the proliferative, migrative and invasive capacity of NSCLC cells *in vivo* and *in vitro*. Mechanistically, based on bioinformatic analysis, RIP assays and rescue experiments, hsa_circ_0072309 was proven to sponge miR-607 and thereby upregulate its target gene fat mass and obesity-associated protein (FTO). Collectively, our study identified a new potential biomarker, hsa_circ_0072309, for NSCLC and established the hsa_circ_0072309/miR-607/FTO axis in tumorigenesis, which shed light on its application in clinical treatment.

## RESULTS

### Hsa_circ_0072309 was upregulated in lung adenocarcinoma

**Figure 1:**
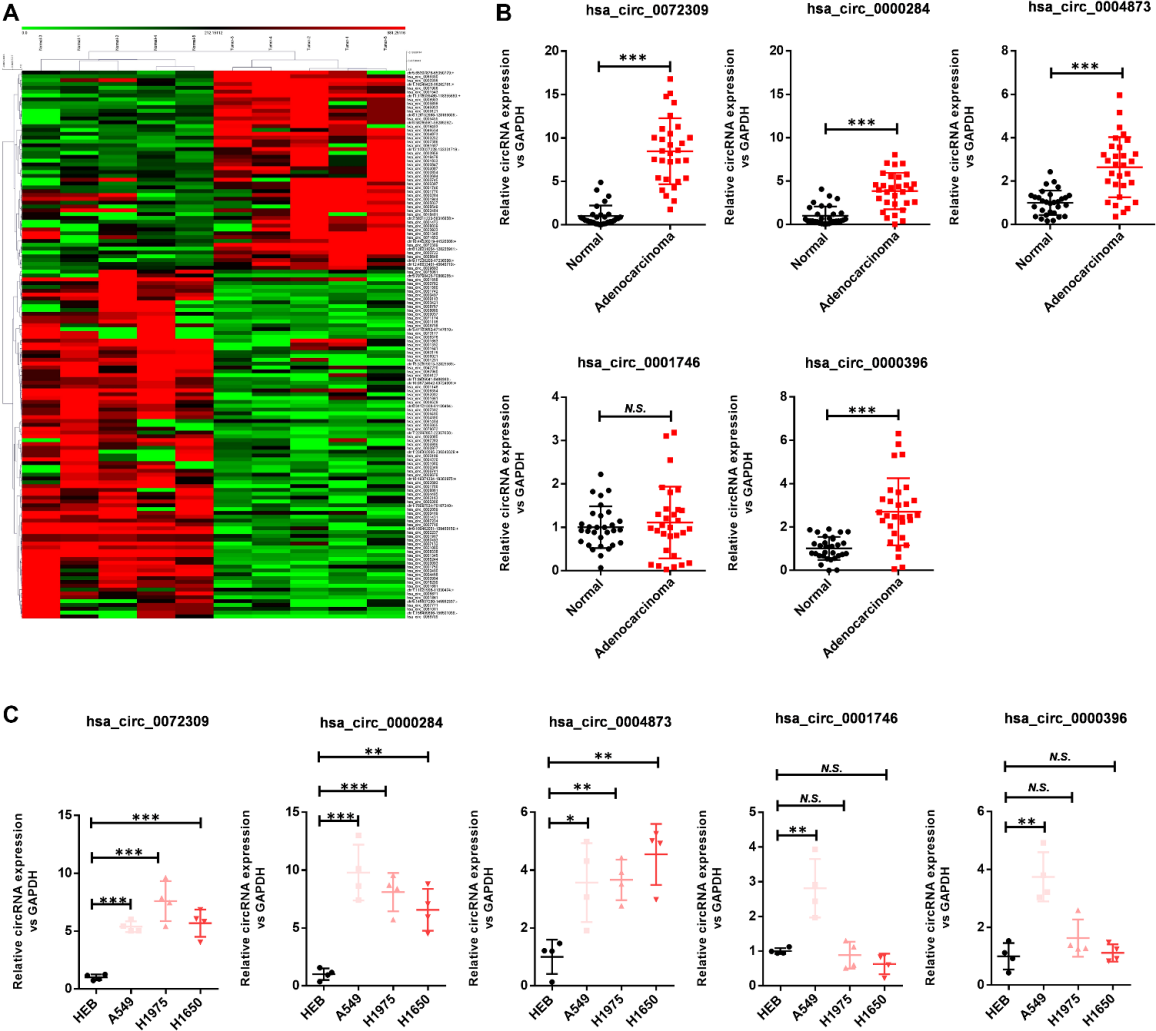
**Hsa_circ_0072309 is upregulated in lung adenocarcinoma.** (**A**) Microarray analysis of human lung adenocarcinoma and adjacent normal tissues (*n* = 5). (**B**) The expression levels of hsa_circ_0072309, hsa_circ_0000284, hsa_circ_0004873, hsa_circ_0001746 and hsa_circ_0000396 in five pairs of human lung adenocarcinoma and adjacent normal tissues. (**C**) The expression levels of hsa_circ_0072309, hsa_circ_0000284, hsa_circ_0004873, hsa_circ_0001746 and hsa_circ_0000396 in the human bronchial epithelial cell line HBE and human lung cancer cell lines (A549, H1975 and H1650). One-way ANOVA followed by Tukey’s multiple comparisons test (**B**). Multiple *t*-test (**C**). N.S.: no significance; ^*^p < 0.05, ^**^p < 0.01, ^***^p < 0.001.

To investigate the roles of circRNAs in NSCLC, we determined the circRNA expression patterns in five pairs of lung adenocarcinoma tissues and corresponding normal tissues after depleting ribosomal RNA and linear RNA molecules to enrich the circRNAs (Figure 1A). As indicated in the heatmap, several circRNAs showed significant abnormal expression in tumors, and these circRNAs were ranked according to the fold changes in expression between the groups. Then, we selected the top five upregulated circRNAs: hsa_circ_0072309, hsa_circ_0000284, hsa_circ_0004873, hsa_circ_0001746 and hsa_circ_0000396. Next, we carried out RT-qPCR analysis to verify the expression of these circRNAs using 30 pairs of lung adenocarcinoma tissues and adjacent normal tissues (Figure 1B). The results indicated that all of these circRNAs were significantly upregulated in lung adenocarcinoma except hsa_circ_0001746. We further confirmed the results in cell lines by using the bronchial epithelial cell line HBE and lung cancer cell lines (A549, H1975 and H1650) (Figure 1C). Likewise, hsa_circ_0072309, hsa_circ_0000284 and hsa_circ_0004873, but not hsa_circ_0000396 and hsa_circ_0001746, were strongly upregulated in lung cancer cell lines. Furthermore, colony formation experiments demonstrated that both hsa_circ_0072309 and hsa_circ_0000284 substantially impaired the proliferative ability of H1975 cells (Supplementary Figure 1). Considering the differential expression levels between adenocarcinoma and normal tissues, we further explored the roles of hsa_circ_0072309 in NSCLC. Together, these findings highlight hsa_circ_0072309 as an oncogene and potential diagnostic and therapeutic marker of NSCLC.

### Hsa_circ_0072309 reduction impaired tumorigenesis in cell lines

**Figure 2:**
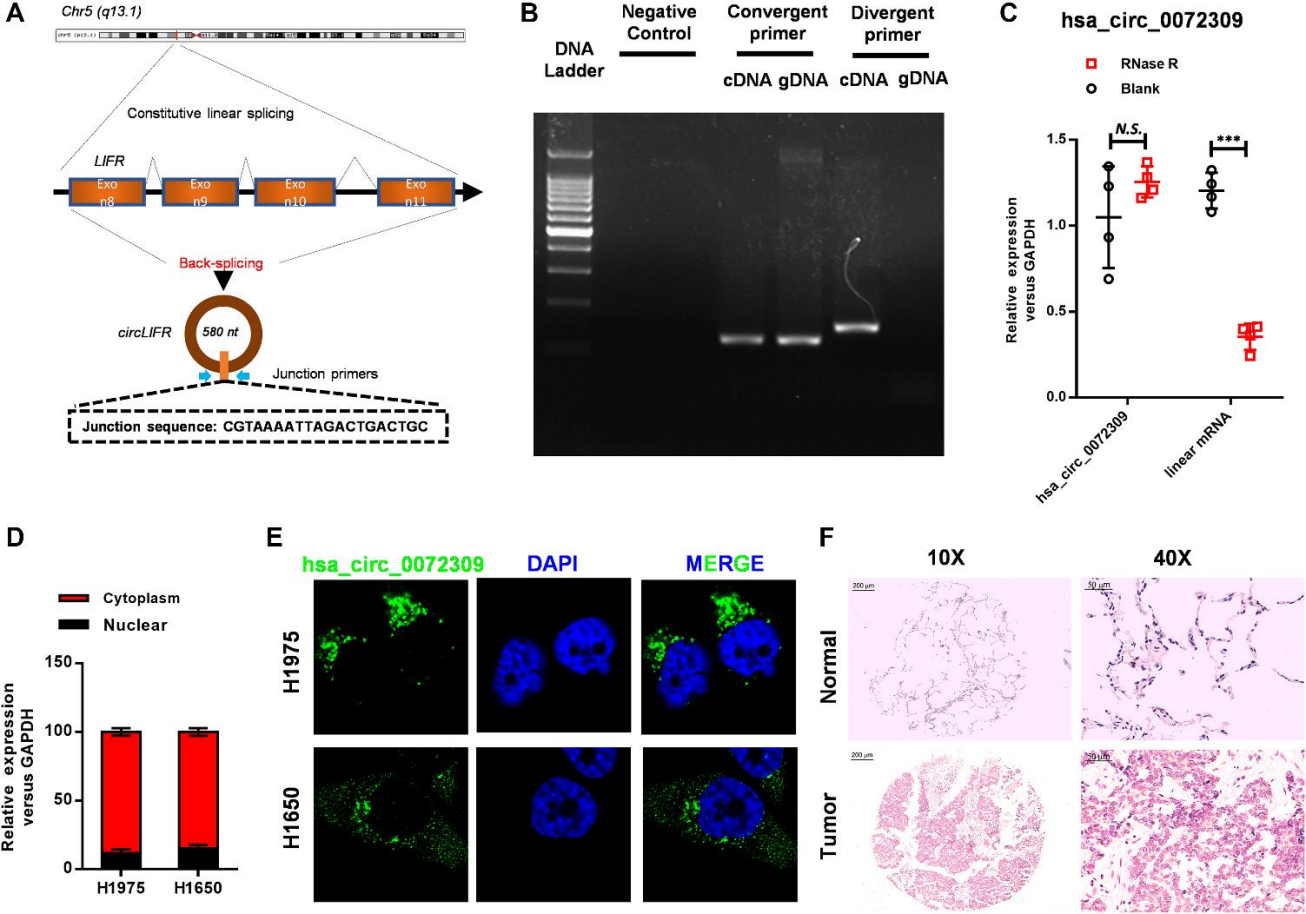
**Hsa_circ_0072309 was reversely looped and located in the tumor cell cytoplasm.** (**A**) The diagram of hsa_circ_0072309. (**B**) Northern blot analyses of hsa_circ_0072309 with convergent primers and divergent primers. (**C**) The expression level of hsa_circ_0072309 and corresponding linear RNA with RNase R digestion. (**D**) The distribution of hsa_circ_0072309 in nuclear and cytoplasmic fractions in H1975 and H1650 cells. (**E**) RNA-FISH analysis of hsa_circ_0072309 in H1975 and H1650 cells. (**F**) ISH against hsa_circ_0072309 in human lung adenocarcinoma and adjacent normal tissues. Multiple *t*-test. N.S.: no significance; ^***^p < 0.001. Scale bars: 50 μm.

Unfamiliar with hsa_circ_0072309, we explored the structure and localization of hsa_circ_0072309 before investigating its biological functions. First, we localized hsa_circ_0072309 on the LIFR gene on chromosome 5 (q13.1), and here, we also named hsa_circ_0072309 as circLIFR. Analyzing the sequence of hsa_circ_0072309, we found that hsa_circ_0072309 was formed by exon back-splicing of exon 8 to exon 11 of the LIFR gene and was 580 nucleotides long (Figure 2A). To identify hsa_circ_0072309, we designed convergent primers and divergent primers based on the junction sequence, and they were used to amplify hsa_circ_0072309 with cDNA samples. The results showed two bands with different sizes. Meanwhile, divergent primers utilized with gDNA samples generated no bands (Figure 2B). It was confirmed that hsa_circ_0072309 was reversely looped. Next, total RNA was separated from NSCLC cell H1975 and digested with RNase R. RT-qPCR analysis was performed, and hsa_circ_0072309, instead of linear mRNA, was the main transcript product in NSCLC cells (Figure 2C). Then, we decided to investigate the subcellular localization of hsa_circ_0072309 in NSCLC cells, which was determined by nuclear/cytoplasmic distribution analyses and FISH assays. The results indicated that hsa_circ_0072309 was mainly located in the cytoplasm (Figure 2D–2E). The ISH assay targeting hsa_circ_0072309 using lung tumor and adjacent normal tissues also showed that hsa_circ_0072309 was mainly located in the cytoplasm (Figure 2F).

**Figure 3:**
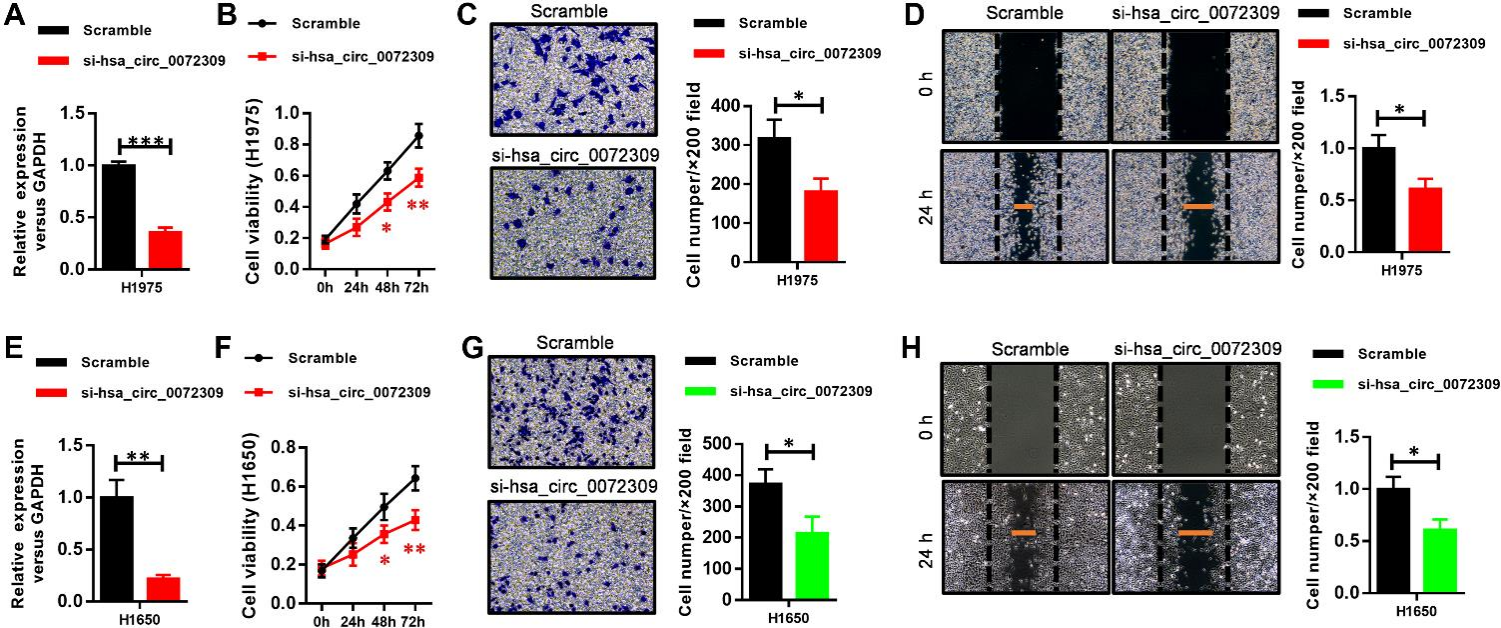
**Hsa_circ_0072309 reduction impaired tumorigenesis in cell lines.** (**A**) Real-time qPCR shows the expression level of hsa_circ_0072309 in lung cancer cells H1975 and its knockdown efficiency. (**B**) Cell proliferation assays of hsa_circ_0072309-knockdown H1975 cells. (**C**) Invasion capability analysis of hsa_circ_0072309-knockdown H1975 cells by Transwell assays. (**D**) Migration ability analysis of hsa_circ_0072309-knockdown H1975 cells by wound-healing assays. (**E**) Real-time qPCR shows the expression level of hsa_circ_0072309 in lung cancer cells H1650 and its knockdown efficiency. (**F**) Cell proliferation assays of hsa_circ_0072309-knockdown H1650 cells. (**G**) Invasion capability analysis of hsa_circ_0072309-knockdown H1650 cells by Transwell assays. (**H**) Migration ability analysis of hsa_circ_0072309-knockdown H1650 cells by wound-healing assays. Quantitative results are indicated in the right panel (**C**–**D**, **G**–**H**). Student’s *t*-test (**A**, **E**). Two-way ANOVA followed by Tukey’s multiple comparisons test (**B**–**D**, **F**–**H**). ^*^p < 0.05, ^**^p < 0.01, ^***^p < 0.001.

To investigate the biological roles of hsa_circ_0072309 in NSCLC, we first reduced the expression level of hsa_circ_0072309 in the NSCLC cell lines H1975 and H1650 using siRNAs, which displayed similar knockdown efficiency (Figure 3A, 3E). We naturally found that decreased expression of hsa_circ_0072309 inhibited cell proliferation of both NSCLC cell lines (Figure 3B, 3F). Moreover, hsa_circ_0072309-KD (knockdown) NSCLC cells showed lower migration and invasion abilities, as measured by Transwell and wound-healing assays, respectively, than control cells (Figure 3C–3D, 3G–3H). Together, these results indicated that hsa_circ_0072309 promoted tumorigenesis in NSCLC cells.

### Hsa_circ_0072309 negatively regulated miR-607 via MRE

**Figure 4:**
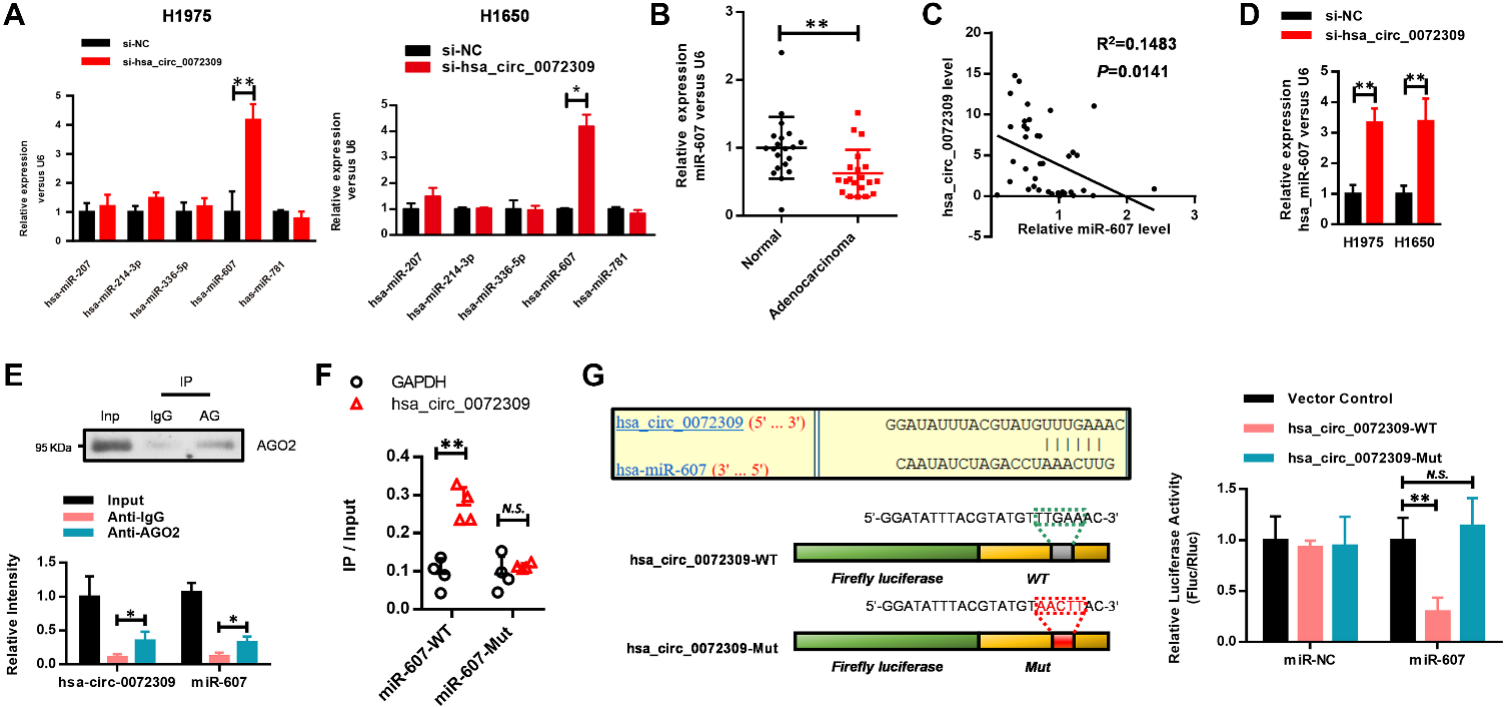
**Hsa_circ_0072309 negatively regulated miR-607 via MRE.** (**A**) Real-time PCR was performed to detect the expression of hsa-miR-207, hsa-miR-214-3p, hsa-miR-336-5p, hsa-miR-607 and hsa-miR-781 in hsa_circ_0072309-downregulated H1975 and H1650 cells. (**B**) The expression level of miR-607 in tumor and normal tissues (*n* = 20). (**C**) The negative correlation of hsa_circ_0072309 with miR-607. (**D**) Real-time qPCR shows the expression level of miR-607 in hsa_circ_0072309-KD H1975 and H1650 cells. (**E**) RNA pulldown analysis of hsa_circ_0072309 against miR-607. (**F**) RNA pulldown was performed to confirm the putative miR-607 binding sites with hsa_circ_0072309. (**G**) A schematic diagram showing the putative miR-607 binding sites with hsa_circ_0072309. Hsa_circ_0072309-WT and hsa_circ_0072309-Mut luciferase reporter vectors were cotransfected with miR-607 or miR-NC and then subjected to luciferase activity analyses. Quantitative results are indicated in the right panel. Two-way ANOVA. N.S.: none significance; ^*^p < 0.05, ^**^p < 0.01.

The above results prompted us to explore the mechanisms underlying the oncogenic functions of hsa_circ_0072309 in NSCLC. Considering that circRNAs competitively sponge miRNAs to regulate biological processes, we established the regulatory relationship of hsa_circ_0072309 and miRNAs. We selected some potential target miRNAs from the circular RNA interactome database to conduct RNA pulldown experiments, and screened out five miRNAs, including hsa-miR-207, hsa-miR-336-5p, hsa-miR-781, hsa-miR-607 and hsa-miR-214-3p (Supplementary Figure 2). The expression changes of the above miRNAs in the si-hsa_circ_0072309-transfected H1975 and H1650 cells were analyzed by real-time PCR. The results indicated that miR-607 expression was most significantly elevated when hsa_circ_0072309 was knocked down (Figure 4A). Analyzing the miRNA expression pattern of 20 pairs of lung adenocarcinoma and adjacent normal tissues, miR-607 stood out with a significantly lower expression level in adenocarcinoma (Figure 4B). We found a negative correlation between the hsa_circ_0072309 and miR-607 levels (Figure 4C), which was further verified in the hsa_circ_0072309-KD H1975 and H1650 cells (Figure 4D).

Next, we tried to explore the underlying mechanism by which hsa_circ_0072309 negatively regulated miR-607. An RNA pulldown assay using AGO2 antibodies was performed to enrich miRNAs, and then, an RT-qPCR assay was used to detect the levels of hsa_circ_0072309 and miR-607. The results showed that hsa_circ_0072309 and miR-607 had similar levels, indicating that hsa_circ_0072309 interacted with miR-607 to a great extent (Figure 4E). To further confirm the interaction between hsa_circ_0072309 and miR-607, we first analyzed the predicted binding sites of miR-607 to hsa_circ_0072309 via https://circinteractome.nia.nih.gov/ and established miR-607-WT and miR-607-Mut constructs with a mutation in the predicted binding site. RNA pulldown assays demonstrated that hsa_circ_0072309 interacted with miR-607-WT instead of miR-607-Mut (Figure 4F). Moreover, luciferase activity assays were used to verify the regulatory mechanism of hsa_circ_0072309 with miR-607. Hsa_circ_0072309 cDNA with the predicted miR-607 binding site was cloned into luciferase reporter vectors (hsa_circ_0072309-WT) and then cotransfected with miR-NC or miR-607. The results demonstrated that when hsa_circ_0072309-WT was cotransfected with miR-607, the luciferase activity was significantly decreased. We also constructed an hsa_circ_0072309-Mut vector with mutations in the miR-607 binding site, and there was no significant difference in the luciferase activity of hsa_circ_0072309-Mut between the miR-NC group and the miR-607 group (Figure 4G). The above results demonstrated that hsa_circ_0072309 negatively regulated miR-607 via MRE.

### Hsa_circ_0072309 promoted tumorigenesis through the miR-607/FTO axis in NSCLC

**Figure 5:**
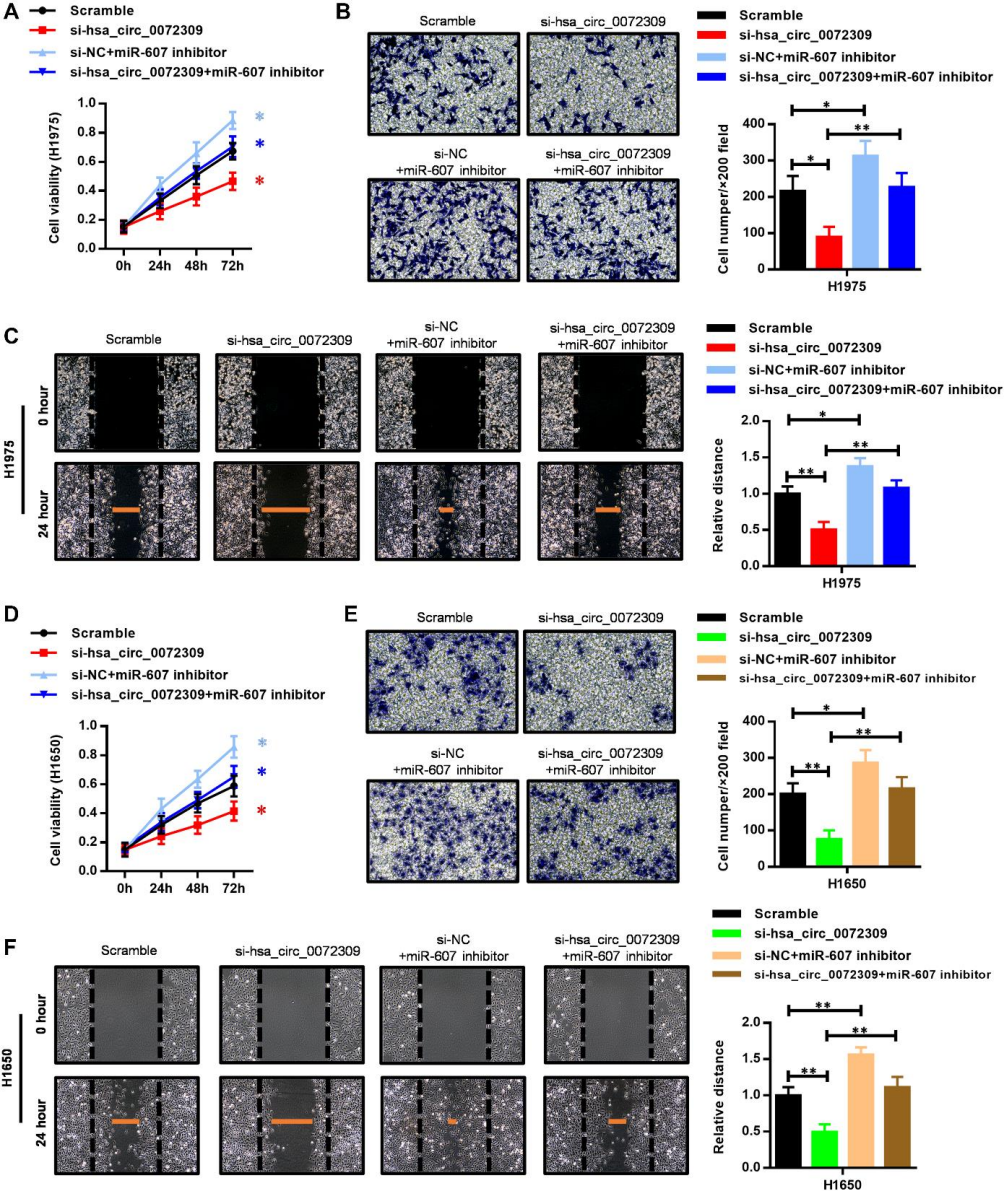
**The miR-607 inhibitor reversed the suppressed cell proliferation, invasion and migration capability caused by hsa_circ_0072309 loss.** (**A**) Cell proliferation assays of control and hsa_circ_0072309-KD H1975 cells with or without miR-607 inhibitor. (**B**) Transwell assays of control and hsa_circ_0072309-KD H1975 cells with or without miR-607 inhibitor. (**C**) Wound-healing assays of control and hsa_circ_0072309-KD H1975 cells with or without miR-607 inhibitor. (**D**) Cell proliferation assays of control and hsa_circ_0072309-KD H1650 cells with or without miR-607 inhibitor. (**E**) Transwell assays of control and hsa_circ_0072309-KD H1650 cells with or without miR-607 inhibitor. (**F**) Wound-healing assays of control and hsa_circ_0072309-KD H1650 cells with or without miR-607 inhibitor. Quantitative results are indicated in the right panel (**B**–**C**, **E**–**F**). Two-way ANOVA followed by Tukey’s multiple comparisons test. ^*^p < 0.05, ^**^p < 0.01.

To gain insight into the mechanism by which the relationship of hsa_circ_0072309 and miR-607 affects tumorigenesis in NSCLC, we transfected miR-607 inhibitor into the hsa_circ_0072309-WT and hsa_circ_0072309-KD H1975 cells. Cell proliferation assays showed that hsa_circ_0072309 ablation decreased cell viability, while the miR-607 inhibitor stimulated cell viability. Moreover, the miR-607 inhibitor successfully reversed the decreased cell viability in the hsa_circ_0072309-KD H1975 cells to the normal level of hsa_circ_0072309-WT cells (Figure 5A). Furthermore, we found that the miR-607 inhibitor suppressed the decrease in invasion and migration caused by the knockdown of hsa_circ_0072309 in H1975 cells (Figure 5B, 5C). Likewise, the regulatory relationship of miR-607 and hsa_circ_0072309 in tumorigenesis was also confirmed in H1650 cells (Figure 5D–5F). The above results indicated that hsa_circ_0072309 negatively regulated miR-607 to promote tumorigenesis in NSCLC cells.

**Figure 6:**
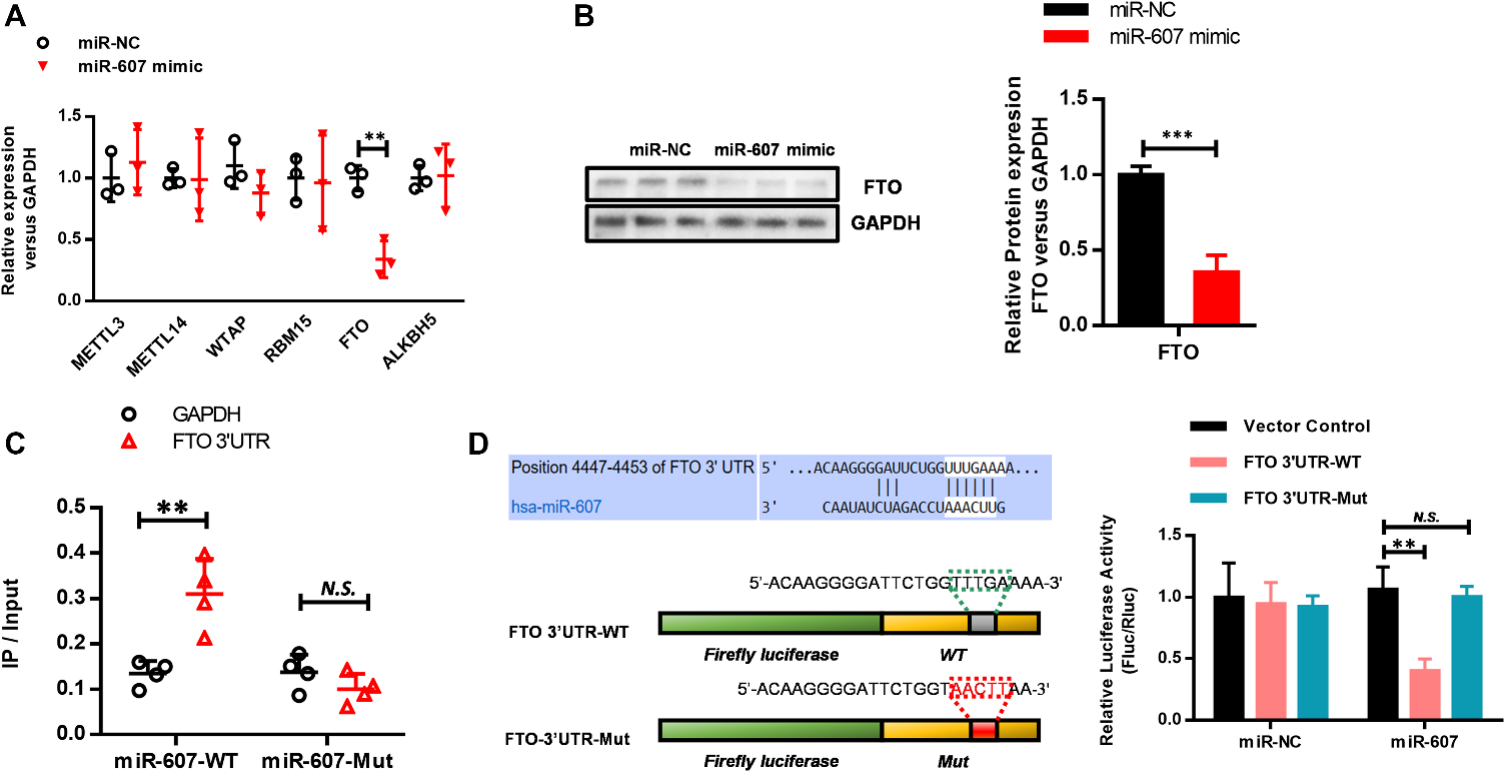
**Hsa_circ_0072309 regulated miR-607 to modulate FTO expression in NSCLC.** (**A**) RT-qPCR was performed to screen FTO as a miR-607 target gene. (**B**) Western blotting analyses of FTO protein levels with or without miR-607 mimic in HEK293 cells. Quantitative results were indicated in the right panel with GAPDH as internal reference. (**C**) RNA pulldown was performed to confirm the putative miR-607 binding sites with FTO 3'UTR. (**D**) A schematic diagram showing the putative miR-607 binding sites with FTO 3'-UTR. FTO 3'-UTR-WT and FTO 3'-UTR-Mut luciferase reporter vectors were cotransfected with miR-607 or miR-NC and then subjected to luciferase activity analyses. Quantitative results are indicated in the right panel. Student’s *t*-test (**B**). Two-way ANOVA (**A**, **C**–**D**). N.S.: no significance; ^**^p < 0.01, ^***^p < 0.001.

Having confirmed that hsa_circ_0072309 negatively regulated miR-607 expression, we determined to find out the functional target gene of miR-607 in NSCLC. Using predictive bioinformatics websites (http://www.targetscan.org/vert_71/), we found several genes containing miR-607 binding sites in the 3'-UTR. To screen out the direct downstream target gene of miR-607, we transfected miR-NC and miR-607 mimic into HEK293 cells separately, and then RT-qPCR analyses were used to detect the expression levels of these candidate target genes. Successfully, we screened FTO as the downstream target gene of miR-607, whose expression level was significantly decreased upon miR-607 mimic transfection (Figure 6A), which was verified by western blotting analyses as well (Figure 6B). To further confirm the regulation of FTO by miR-607, an RNA pulldown assay was performed using miR-607-WT and miR-607-Mut, and the expression level of the FTO 3'-UTR was quantified by RT-qPCR analysis. The results showed that the FTO 3'-UTR interacted with miR-607-WT instead of miR-607-Mut via the binding site of miR-607 to hsa_circ_0072309 (Figure 6C). Next, we performed luciferase assays to confirm the interaction and regulation of miR-607 and FTO. FTO 3'-UTR cDNA (FTO 3'-UTR-WT) and cDNA containing mutations in the predicted binding site of miR-607 (FTO 3'-UTR-Mut) were cloned into luciferase reporter vectors and cotransfected with miR-NC or miR-607. The results indicated that FTO 3'-UTR-WT was greatly reduced by miR-607, while FTO 3'-UTR-Mut showed similar expression levels in the miR-NC group and miR-607 group (Figure 6D). Together, these results demonstrated that hsa_circ_0072309 functioned as an miR-607 sponge to regulate FTO expression.

**Figure 7:**
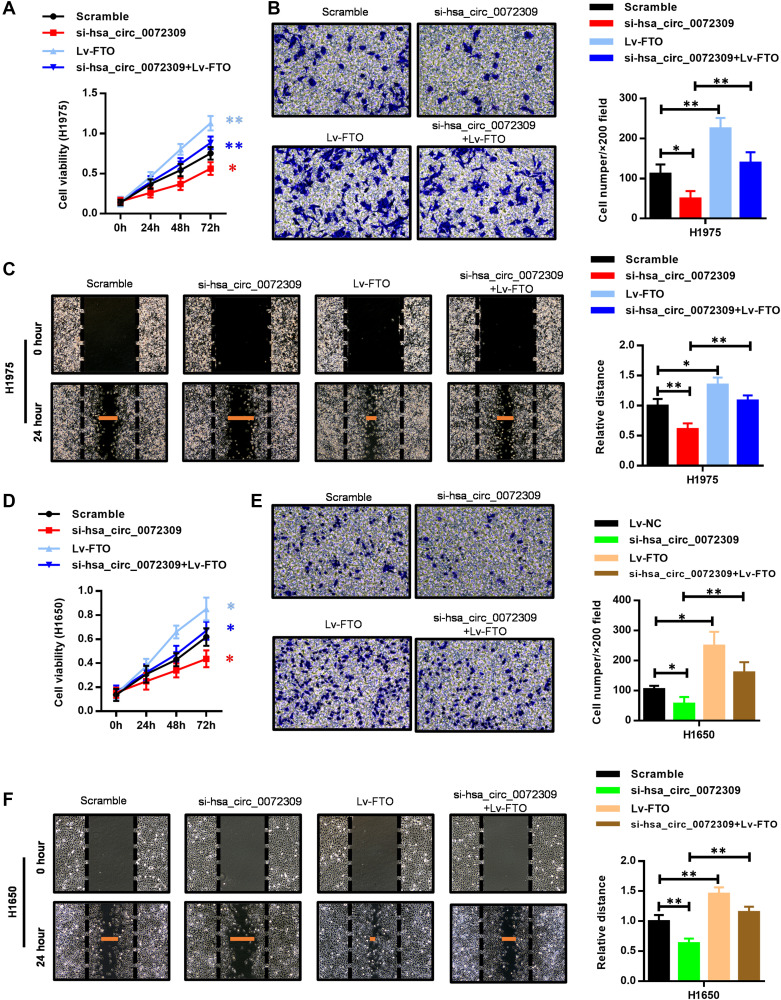
**FTO overexpression reversed the suppressed cell proliferation, invasion and migration capability caused by hsa_circ_0072309 loss.** (**A**) Cell proliferation assays of control and hsa_circ_0072309-KD H1975 cells with or without FTO overexpression. (**B**) Transwell assays of control and hsa_circ_0072309-KD H1975 cells with or without FTO overexpression. (**C**) Wound-healing assays of control and hsa_circ_0072309-KD H1975 cells with or without FTO overexpression. (**D**) Cell proliferation assays of control and hsa_circ_0072309-KD H1650 cells with or without FTO overexpression. (**E**) Transwell assays of control and hsa_circ_0072309-KD H1650 cells with or without FTO overexpression. (**F**) Wound-healing assays of control and hsa_circ_0072309-KD H1650 cells with or without FTO overexpression. Quantitative results are indicated in the right panel (**B–C**, **E–F**). Two-way ANOVA followed by Tukey’s multiple comparisons test. ^*^p < 0.05, ^**^p < 0.01.

**Figure 8:**
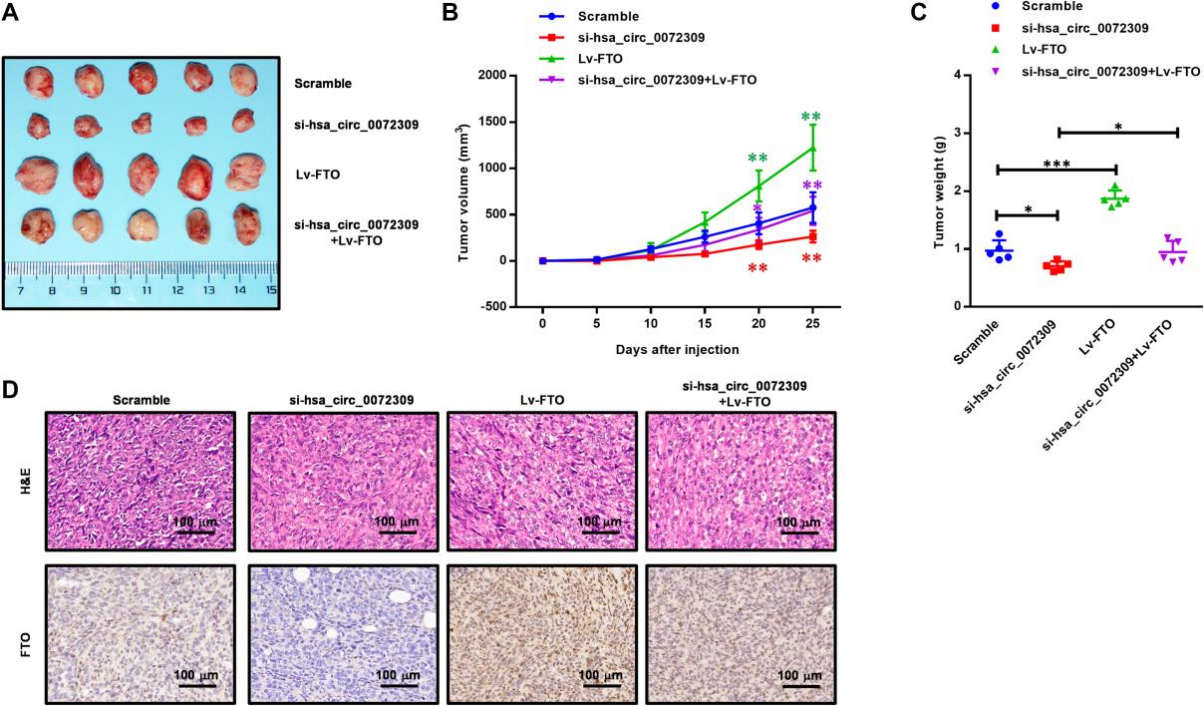
**Hsa_circ_0072309 promoted tumorigenesis through the miR-607/FTO axis in NSCLC.** (**A**) A representative image of the tumor volume of control and hsa_circ_0072309-KD H1975 cells with or without FTO overexpression (*n* = 5). (**B**) Measurement of subcutaneous tumor growth of control and hsa_circ_0072309-KD H1975 cells with or without FTO overexpression (*n* = 5, two-way ANOVA followed by Tukey’s multiple comparisons test. (**C**) Subcutaneous tumors were excised and weighed after mice were sacrificed (*n* = 5, one-way ANOVA followed by Tukey’s multiple comparisons test). (**D**) H&E and FTO staining of subcutaneous tumors of control and hsa_circ_0072309-KD H1975 cells with or without FTO overexpression. ^*^p < 0.05, ^**^p < 0.01. Scale bars: 50 μm. ^*^p < 0.05, ^**^p < 0.01, ^***^p < 0.001.

Having revealed the molecular mechanism of hsa_circ_0072309 with the miR-607/FTO axis, we decided to further confirm its role in regulating tumorigenesis in NSCLC cells (H1975 and H1650). We ectopically expressed FTO in the hsa_circ_0072309-WT and hsa_circ_0072309-KD NSCLC cells. Cell proliferation assays showed that FTO overexpression significantly promoted cell viability in the hsa_circ_0072309-WT cells and reversed the decreased cell viability in the hsa_circ_0072309-KD cells to the normal level (Figure 7A, 7D). Likewise, we also found that FTO overexpression reversed the decreased cell invasion and migration caused by the knockdown of hsa_circ_0072309 in NSCLC cells (Figure 7B–7C, 7E–7F). To validate our genetic findings in cells, we utilized the nude mouse xenograft model. We first knocked down hsa_circ_0072309 in H1975 cells using siRNA to establish hsa_circ_0072309-KD H1975 cells (si-hsa_circ_0072309) and then ectopically expressed FTO in scramble (Lv-FTO) and si-hsa_circ_0072309 (si-hsa_circ_0072309 + Lv-FTO) H1975 cells, which were subcutaneously injected into nude mice. Xenograft assays indicated that hsa_circ_0072309 loss greatly suppressed tumor growth, while FTO overexpression significantly promoted tumor growth (volume and weight) and even successfully reversed the reduced tumor growth (volume and weight) of the hsa_circ_0072309-KD H1975 cells to that of normal tumors (Figure 8A–8C). H&E staining and IHC staining against FTO demonstrated the positive correlation of FTO expression level and tumor malignancy (Figure 8D). Thus, these results demonstrated that hsa_circ_0072309 sponged miR-607 to promote tumorigenesis via the miR-607/FTO axis in NSCLC.

## DISCUSSION

CircRNAs are commonly generated from the exons of protein-coding gene transcripts by RNA splicing and are more abundant than their corresponding linear RNAs [22, 23]. Moreover, many circRNAs show tissue-specific or physiologically specific expression patterns [24–26], indicating crucial regulatory roles of circRNAs in certain kinds of diseases. Recently, scientific studies have investigated the effects and mechanisms of circRNAs in human cancers. Previous studies have suggested that certain circRNAs are abnormally expressed in some types of cancers and regulate cancer progression. For instance, a global reduction in circRNAs was found in CRC tissues compared with adjacent normal tissues. It has been shown that circRNAs accumulate more in nonproliferating cells than in proliferating cells due to their high stability [27]. In gastric cancer, circPVT1 was strongly upregulated and could be used as an independent predictor of better prognosis [28]. In HCC, circMTO1 was found to be significantly decreased and positively correlated with prognosis [19]. However, the aberrant expression of circRNAs in NSCLC is still in its infancy and remains to be elucidated. Therefore, we explored the expression patterns and functions of circRNAs in NSCLC. In the present study, hsa_circ_0072309 was found to be significantly upregulated in NSCLC by circRNA profiling analyses of five pairs of NSCLC and adjacent normal tissues. The expression pattern of hsa_circ_0072309 was further confirmed by RT-qPCR assays in cancer tissues and cell lines.

Considering that hsa_circ_0072309 was significantly upregulated in NSCLC, we explored its biological functions in NSCLC. Our results demonstrated that hsa_circ_0072309 promoted tumorigenesis and metastasis in NSCLC cell lines. To investigate the molecular mechanisms of hsa_circ_0072309 in tumorigenesis, we performed RNA pulldown assays and luciferase activity assays, and the results demonstrated that hsa_circ_0072309 sponged miR-607 to regulate FTO expression.

FTO is one of the only two identified m6A demethylases [29]. As an important type of epigenetic modification, N6-methyladenosine (m6A) was reported to be the most abundant mRNA modification [30, 31], which influenced most of the steps of RNA metabolism and thereby regulated mRNA export from the nucleus to the cytoplasm, RNA decay, mRNA translation and the biogenesis of lncRNAs and miRNAs [32–35]. Moreover, emerging evidence has suggested that m6A regulation is involved in cancer progression [36, 37]. FTO showed oncogenic functions in lung squamous cell carcinoma, enhancing MZF1 expression by decreasing the m6A levels and mRNA stability in MZF1 mRNA transcripts [38]. Likewise, FTO plays a carcinogenic role in acute myeloid leukemia as an m6A demethylase [39]. However, the upstream regulatory axis of FTO was not investigated. Through bioinformatic analyses, we revealed the regulation of FTO by hsa_circ_0072309 via sponging of miR-607, establishing a relationship between circRNAs and the m6A-related mRNA modification factor FTO. The above results indicated a complicated circRNA-miRNA-m6A regulatory network in cancer cells. However, the function of FTO in lung cancer remains to be explored, and we are now focusing on this issue.

Moreover, although the research showed the tumor-driving effects of hsa_circ_0072309 in the process of leading lung cancer, we also believe that there may be other important deregulated circRNAs that participate in the progression of lung cancer, because of the limited tissue samples used for screening. Hence, the deregulated circRNAs in NSCLC pathology still need further investigation.

## MATERIALS AND METHODS

### Human lung adenocarcinoma tissue samples

The 30 pairs of lung adenocarcinoma tissues and paired adjacent normal tissues were acquired from Changzhou Seventh People's Hospital from January 2019 to Mary 2019. NSCLC tissue specimens and corresponding normal tissues were obtained though puncture and packed in liquid nitrogen at -196°C. All experiments in our study were approved by the Ethics Review Committee of Changzhou Seventh People's Hospital. All patients gave informed consent.

### Microarray analysis

TRIzol reagent (Thermo Fisher, USA) was used to extract total RNA from lung adenocarcinoma and adjacent normal tissues according to the manufacturer’s specifications. CircRNAs were enriched by removing linear RNAs with RNase R and then amplified and labeled using an Arraystar Super RNA Labeling Kit (Arraystar, USA). Subsequently, an Arraystar Human circRNA Array applied to hybridization was scanned by an Agilent Scanner G2505C (Agilent, USA). CircRNAs with a fold change of ≥ 2 and a *P*-value of < 0.05 were considered differentially expressed.

### Expression plasmids and siRNA

Full-length human FTO (NM_001080432, Invitrogen, USA) cDNA was duplicated into the pLVX-IRES-Puro vector (Clontech, USA) to generate FTO expression plasmids. The siRNA targeting hsa_circ_0072309 (siRNA sequence: GCAGTCAGTCTAATTTTACG) was obtained from GenePharma (Shanghai, China).

### Cell line culture and reagents

All cells in this study were obtained from ATCC. Human bronchial epithelial HBE cells and human NSCLC H1975 and H1650 cells were cultured in RPMI 1640 medium with 10% fetal bovine serum (FBS, Gibco, USA) and 1% penicillin/streptomycin solution (Gibco, USA). Lentivirus was used to establish individual stable cell lines. SiRNA duplexes specific to hsa_circ_0072309 (100 nmol/L), miR-607 mimic and their negative control (NC) oligonucleotides were transfected into cells with Lipofectamine 3000 (Invitrogen, USA) under the manufacturer’s protocol.

### Cell proliferation assay

Under the manufacturer’s protocol, the CellTiter 96^®^ Non-Radioactive Cell Proliferation Assay (MTT) Kit (Promega, USA) was used to perform the cell proliferation assays. Cells (1 × 10^4^ cells/ml) were seeded into 96-well plates (100 ml/well) and incubated at 37°C in a humidified atmosphere containing 5% CO_2_. Ten milliliters of MTS solution was added to each well and then incubated at 37°C for 2 h. The absorbance at 590 nm for each sample was measured by using a spectrophotometer. All experiments were repeated three times and were performed in triplicate.

### Cell invasion assay

Transwell chambers (8 mm pore size; Corning, USA) were used to perform the Transwell assays for the determination of cell invasion. H1975 and H1650 cells (1 × 105) were resuspended in RPMI 1640 medium without serum (Gibco, USA) and seeded into the upper chamber. RPMI 1640 medium containing 20% FBS was added to the bottom chamber. After 48 h of incubation, cells on the upper side of the chamber were scraped off with cotton swabs. Then, the filters were fixed in 4% paraformaldehyde (PFA, Sigma Aldrich, USA) and stained with 0.1% crystal violet (Beyotime, Shanghai, China) for 15 min and 10 min, respectively. After three washes with phosphate buffered saline (PBS, Gibco, USA), cells invading through the Matrigel were imaged, and the number of cells was counted in five random views using a microscope (Olympus, Japan). Each assay was performed in triplicate.

### Cell migration assay

A wound-healing assay was used to detect cell migration. First, cells were added to 6-well plates. When the confluence of cells reached approximately 90%, a 200 ml pipette tip was used to make the artificial wounds. After 24 h, the wound closure distance was measured by using a microscope. Each assay was performed in triplicate.

### RNA isolation and real-time qPCR

Total RNA was isolated using TRIzol under the instructions of manufacturer. CircRNAs were then enriched by removing linear RNAs with RNase R. First strand cDNA was generated by using Superscript II (Invitrogen, USA), and 1 μg of total RNA was used in reverse transcription. SYBR Green Universal Master Mix reagent (Roche, USA) and primer mixtures were used to conduct real-time qPCR assays. GAPDH was used as a control for circRNAs. The outward-facing primers used for RT-qPCR analysis were purchased from Geneseed (Guangzhou, China) and the primer sequences are shown in Table 1. The primer sequences for hsa_circ_0072309 were shown as below:

Convergent Primer:

F:5'-ATTGCACAGATGATGGATATTT-3';

R: 5'-CAATGCAAACTTCATAATCAGTACC-3'.

Divergent Primer:

F:5'-CACTAAATGAACAAAACGTTTCC-3';

R: 5'-TATAGAAGAAGAAATGTTGATA-3'.

**Table 1:** Primers used for real-time qPCR.

**Genes**	**Forward (5'–3')**	**Reverse (5'–3')**
hsa_circ_0072309	CAAAACCCCTCCTGATGAGA	AATTTACACGAACCGCAAGG
hsa_circ_0000284	GACAGCTACCACAGGATCAA	CCAGCATCTCAAAGACTAAAC
hsa_circ_0004873	GCTCTTGAGTTTGAGGATGG	CATCGCTGGAGAAAAAAGAGG
hsa_circ_0001746	AGAAACATTCACCTTGAAGC	AATCACCCTTCAACACCAGC
hsa_circ_0000396	GTGTCAAGCTTCCATTTTGG	GAGACTTCTTCTACTTTCACG
METTL3	TTGTCTCCAACCTTCCGTAGT	CCAGATCAGAGAGGTGGTGTAG
METTL14	GAACACAGAGCTTAAATCCCCA	TGTCAGCTAAACCTACATCCCTG
WTAP	CTTCCCAAGAAGGTTCGATTGA	TCAGACTCTCTTAGGCCAGTTAC
FTO	AACACCAGGCTCTTTACGGTC	TGTCCGTTGTAGGATGAACCC
ALKBH5	CGGCGAAGGCTACACTTACG	CCACCAGCTTTTGGATCACCA
RBM15	TCCCACCTTGTGAGTTCTCC	GTCAGCGCCAAGTTTTCTCT
GAPDH	GCGGGGCTCTCCAGAACATC	TCCACCACTGACACGTTGGC
MiR-207	GCTGGGAAGGCAAAGGGACGT	TGGTGTCGTGGAGTCG
MiR-214-3p	TCAGTGCATGACAGAACTTGG	TGGTGTCGTGGAGTCG
MiR-336-5p	GGAGACTGATGAGTTCCCGGGA	TGGTGTCGTGGAGTCG
MiR-607	GTTCAAATCCAGATCTATAAC	TGGTGTCGTGGAGTCG
MiR-781	GCCCTGTGGACTCAGTTCTGGT	TGGTGTCGTGGAGTCG
U6	CTCGCTTCGGCAGCACA	AACGCTTCACGAATTTGCGT

### Western blotting analyses

Sodium dodecyl sulfate-polyacrylamide gel electrophoresis (SDS-PAGE) gels were used to extract and separate the total protein of each group. FTO antibody (ab94482, Invitrogen, USA) and AGO2 antibody (ab57113, Cell Signaling Technology, USA) were used at a 1:2000 dilution. GAPDH antibody (5174, 1:2000, Cell Signaling Technology, USA) was used as a loading control.

### Nuclear mass separation

Under the manufacturer’s specifications, the SurePrep™ Nuclear or Cytoplasmic RNA Purification Kit (Fisher BioReagents, USA) was used to separate nuclear and cytoplasmic fractions. Real-time qPCR assays were used to detect the RNA expression levels of hsa_circ_0072309, RNU6-1 and GAPDH. RNU6-1 and GAPDH were used as internal controls.

### RNA-fluorescence *in* *situ* hybridization (RNA-FISH)

Cells were fixed in 4% PFA and then permeabilized with 0.5% Triton X-100 at 4°C for 15 min. Hsa_circ_0072309 probe labeled with digoxigenin (DIG) or the control probe mix was incubated with the cells for 4 h at 55°C. After three brief washes with 2 × saline-sodium citrate for 5 min each time, signals were detected using horseradish peroxidase (HRP)-conjugated anti-DIG secondary antibodies (Jackson, USA). The nucleus was counterstained with 4′,6-diamidino-2-phenylindole (DAPI, Thermo Fisher, USA). A confocal microscope was used to acquire the images. ISH was performed as described elsewhere [40]. The expression level of hsa_circ_0072309 was determined with an Aperio ImageScope V11 (Leica, Mannheim, Germany) and was represented as a positivity value × 100. The sequence of the probe for ISH was 5'-CUGGAAAUUUGAAGCAGUCCUC-3'.

### Luciferase reporter assays

For construction of the hsa_circ_0072309-WT luciferase reporter vector, hsa_circ_0072309 cDNA, which had the predicted miR-607 binding site, was cloned into the pmirGLO vector (Promega, USA). The hsa_circ_0072309-Mut vector was generated by inserting mutant hsa_circ_0072309 with point mutations in the miR-607 binding site. Likewise, the FTO 3'UTR-WT and FTO 3'UTR-Mut luciferase reporter vectors were constructed by cloning wild-type and mutant FTO 3'-UTR fragments into the pmirGLO vector.

Lipofectamine 3000 (Invitrogen, USA) was used to cotransfect miR-607 or miR-NC with the reporter vector into HEK293 cells. The luciferase activity was detected with a Dual Luciferase Reporter Assay System (Promega, USA) according to the manufacturer’s protocol after transfection for 48 h. Each assay was performed in triplicate.

### RNA pulldown

First, 3'-end biotinylated miR-607 and miR-607-Mut were transfected into cells when the final concentration reached 20 nmol/L. After 24 h, the cells were obtained, and streptavidin magnetic beads (Ambion, Life Technologies, USA) were incubated in the cell lysate for RNA pulldown assays. Real-time qPCR assays were used to analyze the abundance of hsa_circ_0072309 or FTO.

### Xenograft tumor model

Five-week-old BALB/c nude mice obtained from Shanghai SLAC Laboratory Animal Center were used for the vivo xenotransplantation assays. The animal experiments were conducted with the permission of the Institutional Animal Care and Use Committee of Changzhou Seventh People's Hospital. H1975 cells were subcutaneously injected into nude mice. There were five mice per group. Tumor volumes were measured every five days. Tumor volumes were estimated by measuring their length and width and calculated using the following equation: V = 0.5 * length * width^2. Approximately one month later, all mice were euthanized, and then, the tumors were resected for weighing and imaging.

### Immunohistochemistry assays

Tumors separated from the nude mice were embedded in paraffin after fixation with 4% PFA. IHC assays were conducted using specific anti-FTO (Invitrogen, USA).

### Statistical analysis

All of the above experiments were carried out using three independent repeated experiments with cells. GraphPad Prism 8.0 (La Jolla, CA, USA) was used for statistical analyses. The results are described as the mean ± SEM. Pearson correlation coefficients were used to determine the correlation between the expression of hsa_circ_0072309 and miR-607. Student’s *t*-test, one-way ANOVA and two-way ANOVA were applied to determine the statistical significance. For all statistical tests, a *P* value < 0.05 was defined as statistically significant.

## Supplementary Materials

Supplementary Figures
