# Supplementary material for: Circular RNA hsa_circ_0072309 promotes tumorigenesis and invasion by regulating the miR-607/FTO axis in non-small cell lung carcinoma
Source: Aging (Albany NY). 2021 Apr 20;13(8):11629–45. doi: 10.18632/aging.202856 (PMC8109101; doi:10.18632/aging.202856)
Supplement: Supplementary Figures [file aging-13-202856-s001.pdf]

SUPPLEMENTARY FIGURES

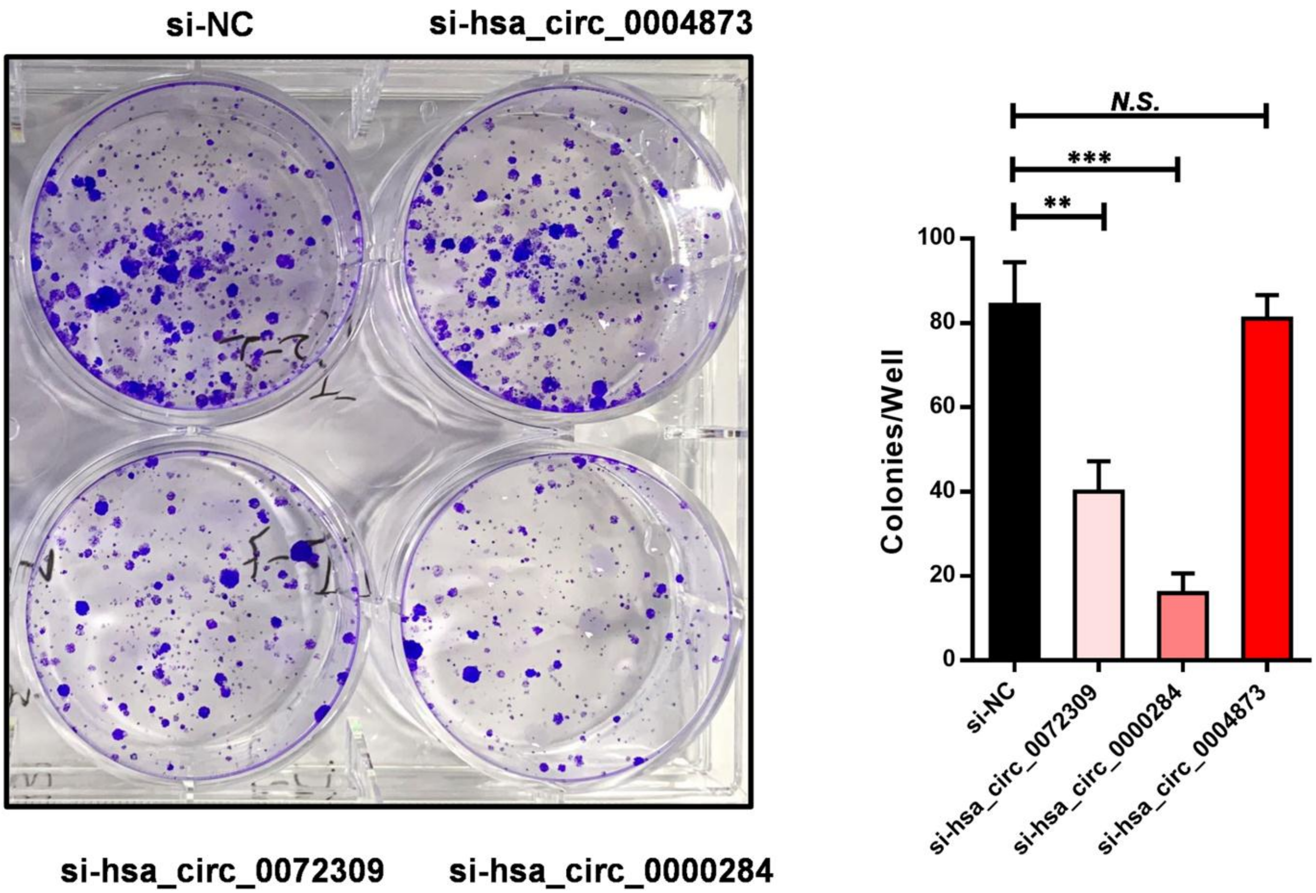

Supplementary Figure 1. Hsa_circ_0072309 and hsa_circ_0000284 knockdown impaired colony formation in H1975 cell lines. Colony formation assay of hsa_circ_0072309-knockdown and hsa_circ_0000284-knockdown H1975 cells. N.S.: no significance; $^{**}p < 0.01$, $^{***}p < 0.001$.

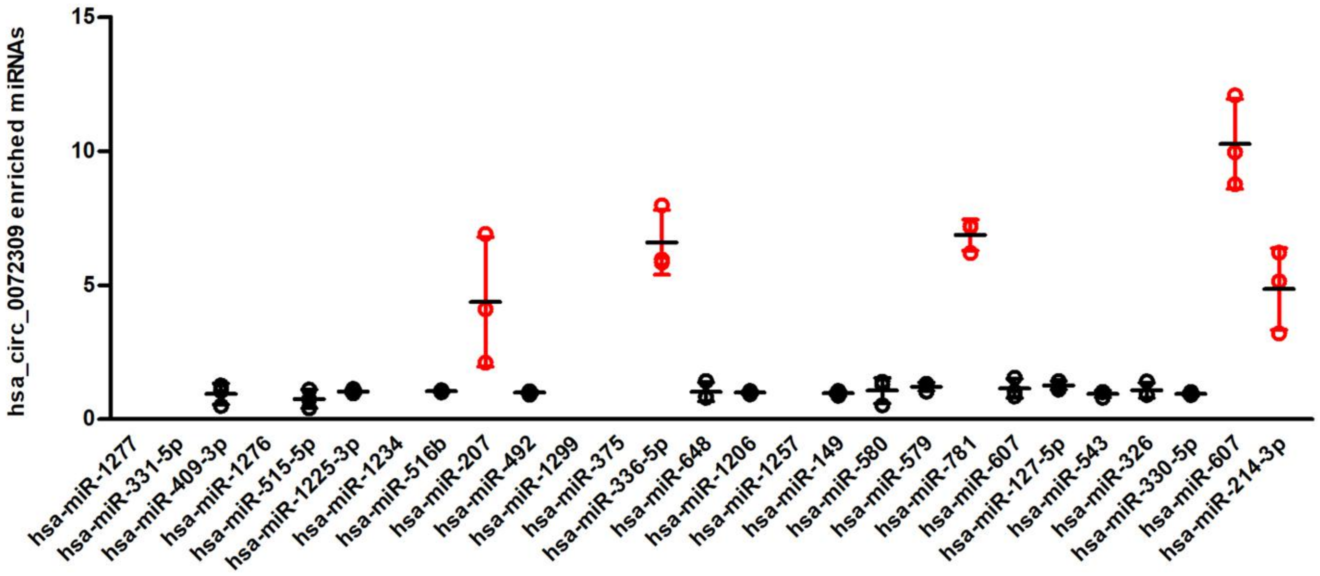

Supplementary Figure 2. Five miRNAs (hsa-miR-207, hsa-miR-214-3p, hsa-miR-336-5p, hsa-miR-607, hsa-miR-781) are enriched in hsa_circ_0072309. RNA pulldown analysis of hsa_circ_0072309 against miRNAs.
